# Retraction Note: Partial differential equations of entropy analysis on ternary hybridity nanofluid flow model via rotating disk with hall current and electromagnetic radiative influences

**DOI:** 10.1038/s41598-025-92440-8

**Published:** 2025-03-07

**Authors:** Khalid Fanoukh Al Oweidi, Faisal Shahzad, Wasim Jamshed, Usman, Rabha W. Ibrahim, El Sayed M. Tag El Din, Afrah M. AlDerea

**Affiliations:** 1 Department of Water Resources Management Engineering, College of Engineering, Al-Qasim Green University, Babylon, Iraq; 2 Department of Mathematics, Capital University of Science and Technology (CUST), Islamabad, 44000 Pakistan; 3 Department of Computer Science, National University of Sciences and Technology, Balochistan Campus (NBC), Quetta, 87300 Pakistan; 4 Department of Mathematics, Mathematics Research Center, Near East University, Near East Boulevard, 99138 Nicosia/Mersin 10, Turkey; 5 Departement of Electrical Engineering, Faculty of Engineering and Technology, Future University, New Cairo, 11835 Egypt; 6 Department of Mathematics, College of Science and Arts, Qassim University, 51951 Al-Badaya, Saudi Arabia

Retraction of: *Scientific Reports* 10.1038/s41598-022-24895-y, published online 30 November 2022

The Editors have retracted this Article as it contains substantial overlap with a previously published article^1^ and several nonstandard phrases. In addition, the Article shows evidence of authorship irregularities. The Authors were unable to provide any response to these concerns and raw data. The Editors therefore no longer have confidence in the reliability of this Article.

The Authors have not responded to any correspondence from the Editors about this retraction.

## References

[1] Khan, M., Ali, W. & Ahmed, J. A hybrid approach to study the influence of hall current in radiative nanofluid flow over a rotating disk. *Appl. Nanosci.* **10**, 5167–5177. 10.1007/s13204-020-01415-w (2020).

